# A Prospective Study of Gynecological Cancer Risk in Relation to Adiposity Factors: Cumulative Incidence and Association with Plasma Adipokine Levels

**DOI:** 10.1371/journal.pone.0104630

**Published:** 2014-08-12

**Authors:** Meei-Maan Wu, Hui-Chi Chen, Chi-Ling Chen, San-Lin You, Wen-Fang Cheng, Chi-An Chen, Te-Chang Lee, Chien-Jen Chen

**Affiliations:** 1 Graduate Institute of Oncology, College of Medicine, National Taiwan University, Taipei, Taiwan; 2 Genomics Research Center, Academia Sinica, Taipei, Taiwan; 3 School of Public Health, Taipei Medical University, Taipei, Taiwan; 4 Graduate Institute of Clinical Medicine, College of Medicine, National Taiwan University, Taipei, Taiwan; 5 Department of Obstetrics and Gynecology, National Taiwan University Hospital, Taipei, Taiwan; 6 Institute of Biomedical Sciences, Academia Sinica, Taipei, Taiwan; Kagoshima University Graduate School of Medical and Dental Sciences, Japan

## Abstract

**Background:**

Associations of obesity and obesity-related metabolic factors (adiposity factors) with uterine corpus cancer (UCC) and ovarian cancer (OVC) risk have been described. Still, a cause-effect relationship and the underlying mediators remain unclear, particularly for low-incidence populations. We aimed to prospectively determine whether adiposity factors could predict the development of UCC and OVC in Taiwanese women. To explore the biological mediators linking adiposity factors to cancer risk, we examined the association of two adipokines, leptin and adiponectin, with the gynecological cancers.

**Methods:**

Totally, 11,258 women, aged 30–65, were recruited into the Community-Based Cancer Screening Program (CBCSP) study during 1991–1993, and were followed for UCC and OVC cases until December 31, 2011. Cox proportional hazard models were used to estimate hazard ratios (HRs). Adiposity factors and risk covariates were assessed at recruitment. Newly-developed cancer cases were determined from data in the government’s National Cancer Registry and Death Certification System. For adipokienes study, a nested case-control study was conducted within the cohort. Baseline plasma samples of 40 incident gynecological cancer cases and 240 age-menopause-matched controls were assayed for adipokines levels.

**Findings:**

There were 38 and 30 incident cases of UCC and OVC, respectively, diagnosed during a median 19.9 years of follow-up. Multivariate analysis showed that alcohol intake (HR = 16.00, 95% = 4.83–53.00), high triglyceride levels (HR = 2.58, 95% = 1.28–5.17), and years of endogenous estrogen exposure per 5-year increment (HR = 1.91, 95% = 1.08–3.38) were associated with increased UCC risk. High body mass index (BMI≥27 kg/m^2^, HR = 2.90, 95% = 1.30–6.46) was associated with increased OVC risk. Analysis further showed an independent effect of adipokines on UCC and OVC risk after adjustment of the risk covariates.

**Conclusion:**

We provided evidence that alcohol intake, high triglyceride levels and long endogenous estrogen exposure increase UCC risk, whereas obesity positively predicts OVC risk. Circulating adipokines may mediate the link of adiposity factors to gynecological cancer risk.

## Introduction

Compared to Western countries, the incidences of non-viral gynecological cancers are relatively low in Asian countries [1], [2]. In 2008, the age-standardized rates were 9.76 and 7.79 per 100,000 for cancer of the uterine corpus (UCC) and ovary (OVC), respectively, among Taiwanese women [3], which represent approximately half the incidences of those seen in Western populations. The low incidences may reflect the differences in etiological factors and/or the prevalence of related factors among Asian women. In westernized countries, both reproductive factors and obesity/overweight were suggested to have a close association with the gynecological cancers (“gynecological cancers” or “UCC and OVC”) [4]. However, the mechanisms underlying the association and additional causes may vary by specific cancer, which, particularly for Asian women, remain to be elucidated.

The most well-established risk factors for UCC are reproductive factors [5]. Factors that increase the development of UCC included early menarche, low parity, late menopause, and exogenous unopposed estrogen exposures, such as the use of hormone therapy. On the other hand, increasing parity and oral contraceptive use decrease the UCC risk. A body mass index (BMI) greater than 25 kg/m^2^ is also a significant risk factor for UCC, increasing the risk by 2.89-fold per 10-unit increment of BMI [6]. This association has been related to a mechanism of hormonal disturbance, including elevated estrogen level [7]. In addition, alcohol intake and serum lipids have been suggested to increase UCC risk [8], [9], but have not been confirmed in other studies [10], [11].

While hormonal etiology is consistently related to UCC risk in almost studies, the association of prolonged hormone exposure with OVC development remains to be established [12], [13]. Aside from a direct involvement of steroid hormones, incessant ovulation leading to cell proliferation has long been proposed as an alternative etiologic hypothesis [14]. Family history with genetic predisposition has also been suggested to contribute to the risk of developing OVC [12], [13]. Among modifiable risk factors, cohort studies evaluating the relationship between obesity and OVC showed only modest or null associations [15], [16], [17]. In these studies, however, the true magnitude of obesity may be masked by the confounding effect from strong family history or the use of menopausal hormone therapy. Other unaddressed questions include whether there is a different effect of abdominal obesity versus overall obesity on OVC risk and whether the strength of the association with obesity is stronger in premenopausal women than in postmenopausal women [15], [16].

As obesity, characterized by excess adipose tissues, has been shown one of the most important risk factors for various cancers, including UCC and OVC, many recent studies have focused on what components of the tissues and how they might relate to carcinogenesis [7]. Adipose tissue-secreted proteins (or adipokines) such as leptin and adiponectin have been implicated as mediators in the pathogenesis linking obesity to cancer risk [18]. High circulating level of leptin and low circulating level of adiponetin have been reported in obese patients [18]. Furthermore, increasing studies demonstrated that hyperleptinemia and hypoadiponectinemia are associated with several malignancies like breast cancer and colorectal cancer [18]. It has also been reported that higher levels of leptin and lower levels of adiponectin were found in the serum of UCC patients than in of control subjects [19], [20]. Most recently, two prospective studies support the results from the case-control studies; yet predominantly included women of European descent [21], [22]. As for OVC, no epidemiological studies have evaluated obesity and circulating adipokines levels in relation to OVC risk.

In the present study, we aimed to identify the risk factors for the development of UCC and OVC by following-up a large community-based women cohort in Taiwan. With the increasing prevalence of obesity/overweight and less frequent use of exogenous steroid hormone in Taiwanese women, the magnitude of fat tissue-related risk factors including obesity and dyslipidemia (or adiposity factors) for each gynecological cancer risk would be expressive. To further investigate the molecules mediating the relationship of adiposity factors to the cancer risk, we examined the associations of two adipokines, leptin and adiponectin, with the development of gynecological cancers. The results of this study should have implications in understanding the causal profiles of both UCC and OVC incidence, and also providing a biological link between adiposity factors and the gynecological cancer risk for Asian women.

## Materials and Methods

### Study Cohort and Baseline Characteristics

This study included female participants of the Community-Based Cancer Screening Program (CBCSP). The enrollment of the CBCSP cohort has been described previously [23], [24]. Briefly, a total of 413,820 female residents, aged 30–65, were invited to participate the CBCSP project from seven townships in Taiwan during the period from January 1991 to December 1992. Among the invited female residents, 11,923 women agreed to participate in a cervical neoplasia screening project (CBCSP-HPV study), including HPV test and cytological examination. A follow-up on the test and examination was conducted between 1993 and 1995 [24]. At the cohort enrollment in 1991–1992, all the participants provided informed consent for a questionnaire interview, physical examination, blood collection, and a follow-up for health status. The 11,923 women enrolled in the CBCSP-HPV study [24] constituted the study subjects of the present study. This study was approved by the Institutional Review Board of the National Taiwan University College of Public Health.

For this study, we focused on lifestyle exposure, adiposity factors and reproductive factors which are potentially associated with the two gynecological cancers in literature. Information on demographic variables, lifestyle exposure (cigarette smoking and alcohol intake), and reproductive factors (age at menarche, parity or number of childbirths, use of contraceptive pills, and age at menopause) were derived from a structured questionnaire interview. Habitual smokers or drinkers were defined as those who either smoked or alcohol-drank at least three times a week for at least half year. Anthropometric measures, including height, weight, and waist and hip circumferences, were determined by physical examination. Overnight fasting blood samples were collected and tested for biochemical variables, such as serum cholesterol and triglycerides.

Among the 11,923 study subjects, 665 were excluded, leaving 11,258 eligible for data analyses. The reasons of exclusion included (1) missing information regarding Personal Identification Number and/or birth date for linking to the records in the government registries (n = 24), (2) cases of study cancer diagnosed prior to or occurring within two years after enrollment (n = 16), and (3) having undergone hysterectomy or oophorectomy prior to or within two years after enrollment (n = 625).

### Follow-up and Determination of Cases

We carried out the follow-up on the cancer and vital status of study subjects by a method of data linkage with the health profiles of the National Cancer Registry and National Death Certification System in Taiwan, respectively. These two nation-wide registry systems were implemented before 1978 in Taiwan with accurate, complete, and updated information [2], [24]. All the eligible study subjects were followed from enrollment until cancer occurrence, deceased from any causes, or the end of follow-up (December 31, 2011), whichever came first. Subjects with a history or subsequently diagnosed of any cancer other than UCC or OVC were not excluded until the defined endpoint of follow-up. The study subjects who left countries because of emigration and of other reasons were treated as censored subjects, and their follow-up periods were nevertheless counted for risk calculation. The proportion of these study subjects was considered to be small. The cancer occurrence in our analyses included incidence and deaths from UCC (ICD, 9^th^ Revision, code 182) and OVC (code 183) with a primary diagnosis.

### Nested Case-Control Study and Adipokine Measurements

To investigate the relationship of adipokines with the risk of gynecological cancer, we conducted a case-control study within the CBCSP women follow-up cohort. We included only those cases occurred as of Dec 31, 2006 because the obtainment of informed consent for adipokine studies was not completed for those occurred after the date. For each case identified during the follow-up, 6 control subjects, matched for age (±3 years) and menopausal status, were selected from among those subjects who were free of gynecological cancers at recruitment and also by the date Dec 31, 2006. Five µl of plasma for each subject were retrieved from frozen stock and assayed for leptin and adiponectin levels. The leptin and adiponectin levels in plasma were determined by a method of immunoassay according to the manufacturer’s protocol (B-Bridge International, San Jose, CA). All the cases along with the 582 candidate controls (Details about the candidate controls were described in the Statistical Analysis) were analyzed within the same laboratory batch with 90 samples per testing assay. The technician who performed the assays was blinded to the case control status of the samples. The intra-assay and inter-assay coefficients of variation (CV) for leptin assay were <6% and <9%, respectively. The corresponding CV values for adiponectin assay were <6% and <8%, respectively.

### Statistical Analysis

We calculated incidence rates by 5-year age groups for each cancer, allowing subjects to contribute time to two cancer sites. Baseline descriptive characteristics were examined by cancer site. Cigarette smokers or drinkers of alcohol were defined as those who had at least 4 days a week for at least 6 months. BMI value was calculated as weight (kg) divided by the square of height (m). Two indicators of abdominal obesity, waist circumference and waist-to-hip ratio (WHR, waist divided by hip circumstances) were considered. Use of contraceptives was defined as having ever used contraceptive pills. To calculate total years of endogenous estrogen exposure, we first counted the difference in years between the age at menarche and the age at interview for pre-menopausal women or the age at menopause for postmenopausal women; we then subtracted the years calculated from the product of number of childbirths multiplied by 10 months and divided by 12 months to achieve the total years of endogenous estrogen exposure.

To examine the association of each risk factor at enrollment with subsequent cancer risk, we calculated the hazard ratio (HR) derived from Cox proportional hazards models using attained age during follow-up as the time axis and stratification of birth cohort in 5-year calendar periods [25]. All study variables were categorized except for age and years of endogenous estrogen exposure. BMI was categorized into <23, 23–27, and ≥27 kg/m^2^. For Asian populations, the points of public health action recommended by the World Health Organization are 23 kg/m^2^ (representing increased risk), and 27.5 kg/m^2^ (representing high risk) [26]. In order to have an adequate number of samples in category-specific parameter estimates for data analysis, we arbitrarily chose 27 rather than 27.5 kg/m^2^ in the subsequent analyses. Waist circumstance and WHR were categorized into binary data. The respective cut-off points were 80 cm and 0.82. For serum lipids, we used the following categories: for total cholesterol ≥200 versus <200 mg/dL, and for triglycerides ≥150 versus <150 mg/dL. We chose these cutoff points for serum lipids according to the report of National Cholesterol Education Program Guidelines [27] (also revisit www://heart.org). The total cholesterol level should be: <200 mg/dL normal blood cholesterol, 200–239 mg/dL borderline-high, ≥240 mg/dL high cholesterol. The corresponding values for the classification of triglyceridemia are <150, 150–199, and ≥200 mg/dL. In order to have an adequate sample number in parameter estimates for categorical data analysis of both cancers, we presented the results using two classes, unless otherwise indicated. Women having years of endogenous estrogen exposure <20 were combined and used as the reference category. The relative hazard associated with years of endogenous estrogen exposure was estimated by 5-year increments throughout an individual’s lifetime.

In multivariate analyses, we used Cox regression models to estimate the hazard ratios associated with covariates based on their independent association with gynecological risk in prior analysis. The time scale for multivariate Cox models and the coding of adjustment variables were defined as previously. To estimate the proportion of cases that could be prevented if all women in the population were shifted to the low risk reference category, we calculated population attributable fractions (PAF) for risk factors, which were essentially modifiable for the attempt of intervention [28]. By definition, a category-specific PAF is estimated as ∑pd_i_((HR_i_-1)/HR_i_), where HR_i_ is the adjusted hazard ratio for the ith exposure category and pd_i_ represents the proportion of total cases exposed to the modifiable risk factor.

Lifetime (35–80 years old) cumulative incidence of each cancer was estimated (1-survival) probability using the Cox proportional hazards model for producing the survival function estimates. Attained age during follow-up was used as the time scale. Meanwhile, ENTRY = age at enrollment to the MODEL statement was added to specify the left truncation time for all study subjects. To examine a potential difference in cancer risk at attained age during follow-up for study subjects who were elevated BMI (≥27 kg/m^2^) versus normal BMI (<27 kg/m^2^), we repeated the analysis by adding the BMI variable in the STRATA statement of the Cox model.

For the case-control study, leptin and adiponectin tertiles were calculated according to the baseline adipokines levels of a representative subcohort (n = 582) which has been serving as the comparison group of an ongoing breast cancer case-control study in Taiwan (unpublished data). Adipokines or BMI data were not available for 8 study subjects. Twenty-eight of the remaining 574 subjects fulfill the exclusion criteria as indicated previously for the follow-up study and therefore were excluded from the current analysis, leaving final sample size 546. These subjects constituted the candidate controls of the present nested case-control study. Figure S1 demonstrates the relationship between adipokine tertiles and BMI levels at enrollment in this subcohort, of which indicates a positive correlation of leptin and a negative correlation of adiponectin levels with baseline BMI (*p*<0.01 for both adipokines). To examine whether the baseline adipokines independently associated with the development of gynecological cancer, unconditional logistic regression analysis was used to calculate the odds ratios (OR) in the study subjects with high adipokine levels (the second and the highest tertiles), as compared with those in the lowest tertile 1.

All statistical analyses were performed using SAS win8e (SAS Institute, Cary, NC), and a *p*<0.05 was considered significant.

## Results

During a median follow-up of 19.9 years, 38 and 30 cases were diagnosed of UCC and OVC, respectively, in the cohort of 11,258 women. As shown in Table 1, the women in the 40–49 year age group had the highest incidence of UCC cancer, with 21.9 cases per 100,000 person-years. The overall incidence of UCC was 17.7 per 100,000 person-years, and the mean age of diagnosis was 56.3 years (Table 2). OVC occurred most frequently among women 50–59 year age group at enrollment, with 21.7 cases per 100,000 person-years. The total incidence of OVC was 14.0, and the average age at diagnosis was 61.5 years (Table 2).

**Table 1:** Incidences of uterine corpus cancer (UCC) and ovarian cancer (OVC) by age groups among 11,258 participants out of the CBCSP-HVP cohort, 1991–2011.

Age group, year	No. of person-years	UCC		OVC	
		Cases	IR^a^ (×10^5^)	Cases	IR (×10^5^)
<40	71,209	14	19.66	8	11.23
40–49	59,467	13	21.86	5	8.41
50–59	64,510	9	13.95	14	21.70
≥60	19,899	2	10.05	3	15.08
Total	215,085	38	17.67	30	13.95

a IR: incidence rate.

**Table 2:** Baseline characteristics of 11,258 study subjects by gynecological cancer and without the cancers.

Characteristics	Without the cancers (n = 11,190)	UCC (n = 38)	OVC (n = 30)
Age at enrollment, year	46.6±9.8	44.3±8.5	49.3±9.9
Age at diagnosis, year		56.3±8.6	61.5±8.6
Junior high school or above	3256 (29.1)	8 (21.1)	9 (30.0)
Cigarette smoking, n (%)	108 (1.0)	0 (0.0)	0 (0.0)
Alcohol intake, n (%)	63 (0.6)	3 (7.9)^a^	0 (0.0)
Years of alcohol intake			
<10	28 (59.6)	1 (33.3)	0 (0.0)
10–19	11 (23.4)	2 (66.7)	0 (0.0)
≥20	8 (17.0)	0 (0.00)	0 (0.0)
Waist-to-hip ratio	0.81±0.07	0.81±0.06	0.81±0.07
Total cholesterol, mg/dL	185.2±42.9	187.0±44.6	195.8±32.0
Triglycerides, mg/dL	95 (66–149)	107 (68–209)^a^	108 (64–177)
Age at menarche, year	15.2±1.6	14.9±1.6	15.2±1.8
≤13	2264 (20.2)	8 (21.1)	8 (26.7)
14	1898 (17.0)	10 (26.3)	3 (10.0)
≥15	7028 (62.8)	20 (52.6)	19 (63.3)
No. of childbirths	3.9±1.6	3.4±1.3	4.1±1.7
0 (nulliparous)	36 (0.4)	0 (0.0)	0 (0.0)
1–3	4852 (47.7)	21 (58.3)	9 (32.1)
≥4	5278 (51.9)	15 (41.7)	19 (67.9)
Use of contraceptives, n (%)	3180 (30.9)	10 (27.0)	4 (14.3)
Endogenous estrogen exposure, year	25.3±6.2	26.4±6.0^a^	28.1±6.5

UCC: Uterine corpus cancer; OVC: Ovarian cancer.

Triglyceride value is given as median (interquartile range), and the other values are given as mean±SD or number (%).

a Age-adjusted *p*-value <0.05, compared with the participants without the cancers.

Table 2 shows the characteristics of study subjects at the time of enrollment grouped by gynecologic cancer site and without the cancers after follow-up. The mean age at enrollment was 44.3 (±8.5) years old for women in the UCC group and 49.3 (±9.9) for the OVC group; the difference between these two cancer groups was significant (age-adjusted *p* = 0.028). Women in the UCC group or the OVC group had a relatively low educational level compared to the women without the cancers, but the differences were not statistically significant. Neither cancer group included cigarette smokers. Compared to women without the cancers, a high proportion of women in the UCC group reported alcohol intake (0.6% versus 7.9%, age-adjusted *p*<0.001). High triglyceride levels were observed among the women of the UCC group (age-adjusted *p* = 0.013) at the time of enrollment. Years of endogenous estrogen exposure was significantly higher in the UCC group (age-adjusted *p* = 0.002), but not in the OVC group, as compared with without the cancers group after accounting for age. Other hormone-related factors, including age at menarche, number of child births, and the use of contraceptives showed no statistically significant difference for either cancer group versus without the cancers group.

An analysis of the follow-up study for UCC risk revealed that women alcohol-drinkers had a highly increased UCC risk (HR = 14.64, 95% = 4.48–47.82) as compared with women who were nondrinkers (Table 3). Women who had triglyceride levels ≥150 mg/dL was found to be associated with a 2.44-fold UCC risk (HR = 2.44, 95% = 1.25–4.77) compared with women having triglyceride levels <150 mg/dL. We also identified a 1.94-fold risk (HR = 1.94, 95% = 1.11–3.41) of UCC for those women per 5-year increment in years of endogenous estrogen exposure. An analysis of follow-up study for OVC risk revealed that women with a BMI of ≥27 kg/m^2^ had a more than 2-fold risk (HR = 2.46, 95% = 1.14–5.30) as compared with women with a BMI<27 kg/m^2^. The other adiposity factors or reproductive factors were not found to be significantly associated with subsequent OVC risk in this women cohort.

**Table 3:** Hazard ratio estimates of sociodemographic, adiposity and reproductive factors by gynecologic cancer for the 11,258 study subjects, 1991–2011.

	Without the cancers (n = 11,190)	Uterine corpus cancer (UCC) (n = 38)		Ovarian cancer (OVC) (n = 30)	
Characteristics	No. (%)	No. (%)	HR (95% CI)^a^	No. (%)	HR (95% CI)^a^
*Sociodemographic factors*					
Educational level					
Without or elementary school	7927 (70.9)	30 (79.0)	(Referent)	21 (70.0)	(Referent)
Junior high school or above	3256 (29.1)	8 (21.1)	0.51 (0.22–1.19)	9 (30.0)	1.46 (0.59–3.63)
Alcohol intake					
No	11076 (99.4)	35 (92.1)	(Referent)	30 (100.0)	(Referent)
Yes	63 (0.6)	3 (7.9)	14.64 (4.48–47.82)***	0 (0.00)	0.00 (0.00-N.A.)
*Adiposity factors*					
Body mass index, kg/m^2^					
<23	4548 (40.9)	17 (44.7)	(Referent)	10 (34.5)	(Referent)
23–26	4456 (40.0)	13 (34.2)	0.81 (0.39–1.69)	8 (27.6)	0.77 (0.30–1.98)
≥27	2129 (19.1)	8 (21.1)	1.12 (0.47–2.64)	11 (37.9)	2.15 (0.88–5.26)
Body mass index, kg/m^2^					
<27	9004 (80.9)	30 (79.0)	(Referent)	18 (62.1)	(Referent)
≥27	2129 (19.1)	8 (21.1)	1.24 (0.56–2.72)	11 (37.9)	2.46 (1.14–5.30)*
Waist circumference, cm					
<80	7061 (63.4)	26 (68.4)	(Referent)	16 (55.2)	(Referent)
≥80	4070 (36.6)	12 (31.6)	0.98 (0.47–2.01)	13 (44.8)	1.22 (0.56–2.68)
Waist to hip ratio					
<0.82	6692 (60.1)	24 (63.2)	(Referent)	16 (55.2)	(Referent)
≥0.82	4438 (39.9)	14 (36.8)	1.15 (0.57–2.33)	13 (44.8)	1.02 (0.46–2.25)
Total cholesterol, mg/dL					
<200	7496 (67.5)	23 (60.5)	(Referent)	18 (62.1)	(Referent)
200–239	2510 (22.6)	11 (29.0)	1.64 (0.79–3.42)	8 (27.6)	1.13 (0.48–2.65)
≥240	1107 (10.0)	4 (10.5)	1.54 (0.52–4.57)	3 (10.3)	0.90 (0.26–3.13)
Total cholesterol, mg/dL					
<200	7496 (67.5)	23 (60.5)	(Referent)	18 (62.1)	(Referent)
≥200	3617 (32.6)	15 (39.5)	1.62 (0.83–3.16)	11 (37.9)	1.06 (0.49–2.30)
Triglyceride, mg/dL					
<150	8346 (75.1)	23 (60.5)	(Referent)	20 (69.0)	(Referent)
150–199	1252 (11.3)	5 (13.2)	1.70 (0.64–4.51)	4 (13.8)	1.17 (0.40–3.47)
≥200	1512 (13.6)	10 (26.3)	3.15 (1.46–6.78)**	5 (17.2)	1.14 (0.42–3.12)
Triglyceride, mg/dL					
<150	8346 (75.1)	23 (60.5)	(Referent)	20 (69.0)	(Referent)
≥150	2764 (24.9)	15 (39.5)	2.44 (1.25–4.77)**	9 (31.0)	1.16 (0.52–2.60)
*Reproductive factors*					
Age at menarche, year					
<15	4162 (37.2)	18 (47.4)	(Referent)	11 (36.7)	(Referent)
≥15	7028 (62.8)	20 (52.6)	0.69 (0.36–1.31)	19 (63.3)	0.90 (0.42–1.93)
No. of childbirths					
<4	4888 (48.1)	21 (58.3)	(Referent)	9 (32.1)	(Referent)
≥4	5278 (51.9)	15 (41.7)	0.73 (0.34–1.54)	19 (67.9)	1.60 (0.62–4.14)
Use of contraceptives					
No	7128 (69.2)	27 (73.0)	(Referent)	24 (85.7)	(Referent)
Yes	3180 (30.9)	10 (27.0)	0.76 (0.37–1.59)	4 (14.3)	0.42 (0.14–1.22)
Endogenous estrogen exposure, year					
<20	2436 (24.0)	5 (13.9)	(Referent)	4 (14.3)	(Referent)
5-year increment	7730 (76.0)	31 (86.1)	1.94 (1.11–3.41)*	24 (85.7)	1.72 (0.99–2.99)

a Hazard ratio (HR) and confidence interval (CI) were derived from Cox proportional hazard models using attained age during follow-up as the time axis and stratification of birth cohort in 5-year calendar periods. N.A.: not available.

*p<0.05; **p<0.01; ***p<0.001.

As shown in Table 4 the multivariate regression analysis, simultaneous adjustment for alcohol intake (for UCC group only), BMI value, triglyceride level, and years of endogenous estrogen exposure did not substantially alter the initial results shown in Table 3. Alcohol intake, triglycerides, and years of endogenous estrogen exposure were positively associated with UCC risk, while BMI was positively associated with OVC risk. Attributable fraction analyses for modifiable risk factors showed that 7.4% of total UCC cases would be prevented if alcohol intake was eliminated, and 24.2% of UCC cases would be eliminated if triglyceride levels were lowered to <150 mg/dL in these women. In addition, 24.8% of OVC cases reduction would be achieved following obesity elimination in this women cohort.

**Table 4:** Multivariate model of risk factors for gynecological cancer for the 11,258 study subjects, 1991–2011.

	Uterine corpus cancer (UCC)			Ovarian cancer (OVC)		
Characteristics	HR (95% CI)^a^	*P*-value	PAF^b^	HR (95% CI)	*P*-value	PAF
Alcohol intake						
Yes vs. no	16.00 (4.83–53.00)	<0.001	0.074			
Body mass index, kg/m^2^						
≥27 vs. <27	1.08 (0.48–2.41)	0.849	0.016	2.90 (1.30–6.46)	0.009	0.248
Triglyceride, mg/dL						
≥150 vs.<150	2.58 (1.28–5.17)	0.008	0.242	0.88 (0.37–2.09)	0.778	0.000
Endogenous estrogen exposure, year						
5-year increment	1.91 (1.08–3.38)	0.026	(N.A.)	1.53 (0.87–2.69)	0.140	(N.A.)

a Hazard ratio (HR) and confidence interval (CI) were derived from Cox proportional hazard models using attained age during follow-up as the time axis and stratification of birth cohort in 5-year calendar periods.

b PAF: population attributable fraction of total cases that would be reduced if a modifiable risk factor were eliminated from the study cohort. The formula for PAF estimation is presented in Methods section. N.A.: not applied.

**Figure 1:**
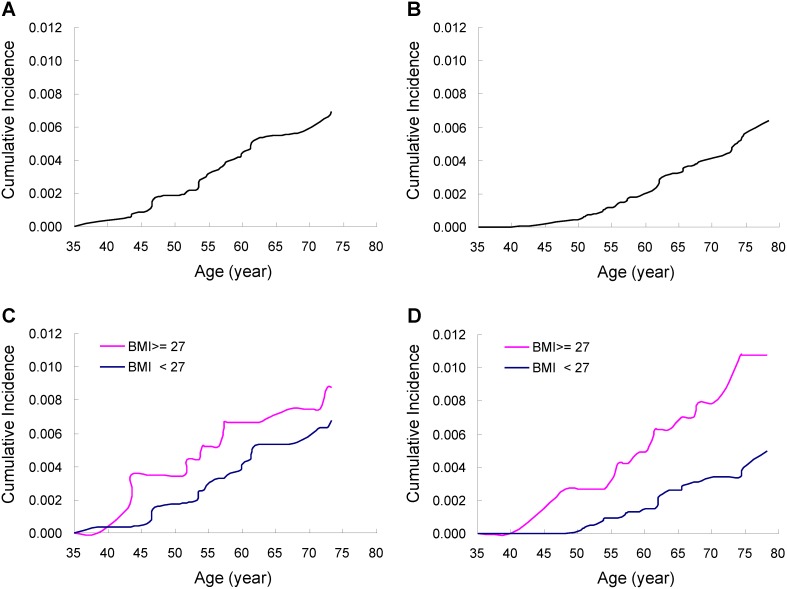
Cumulative incidence of gynecological cancers on attained age during follow-up. **A.** Patients of uterine corpus cancer (UCC, n = 38). **B.** Patients of ovarian cancer (OVC, n = 30). **C.** Patients of UCC for body mass index (BMI) ≥27 kg/m^2^ (n = 8) and <27 kg/m^2^ (n = 30). **D.** Patients of OVC for BMI≥27 kg/m^2^ (n = 11) and <27 kg/m^2^ (n = 18). One patient of ovarian cancer had missing data on BMI.

Figure 1 shows the respective lifetime (35–80 years old) cumulative incidence of each gynecological cancer from the time of enrollment. As shown in the figure, the risk of developing UCC (Figure 1A) and OVC (Figure 1B) among Taiwanese women by the age of 80 years were similar, approximating 0.69% and 0.64%, respectively. Upon further analysis stratifying by BMI level, we found that the effect of obesity was observed throughout an individual’s lifetime for both gynecological cancers. By the age of 80 years, the respective UCC risks for BMI≥27 kg/m^2^ and BMI<27 kg/m^2^ were 0.87% and 0.68% (Figure 1C). The respective estimates of OVC risk by the age of 80 years for BMI≥27 kg/m^2^ and BMI<27 kg/m^2^ were 1.07% and 0.50% (Figure 1D).

**Figure 2:**
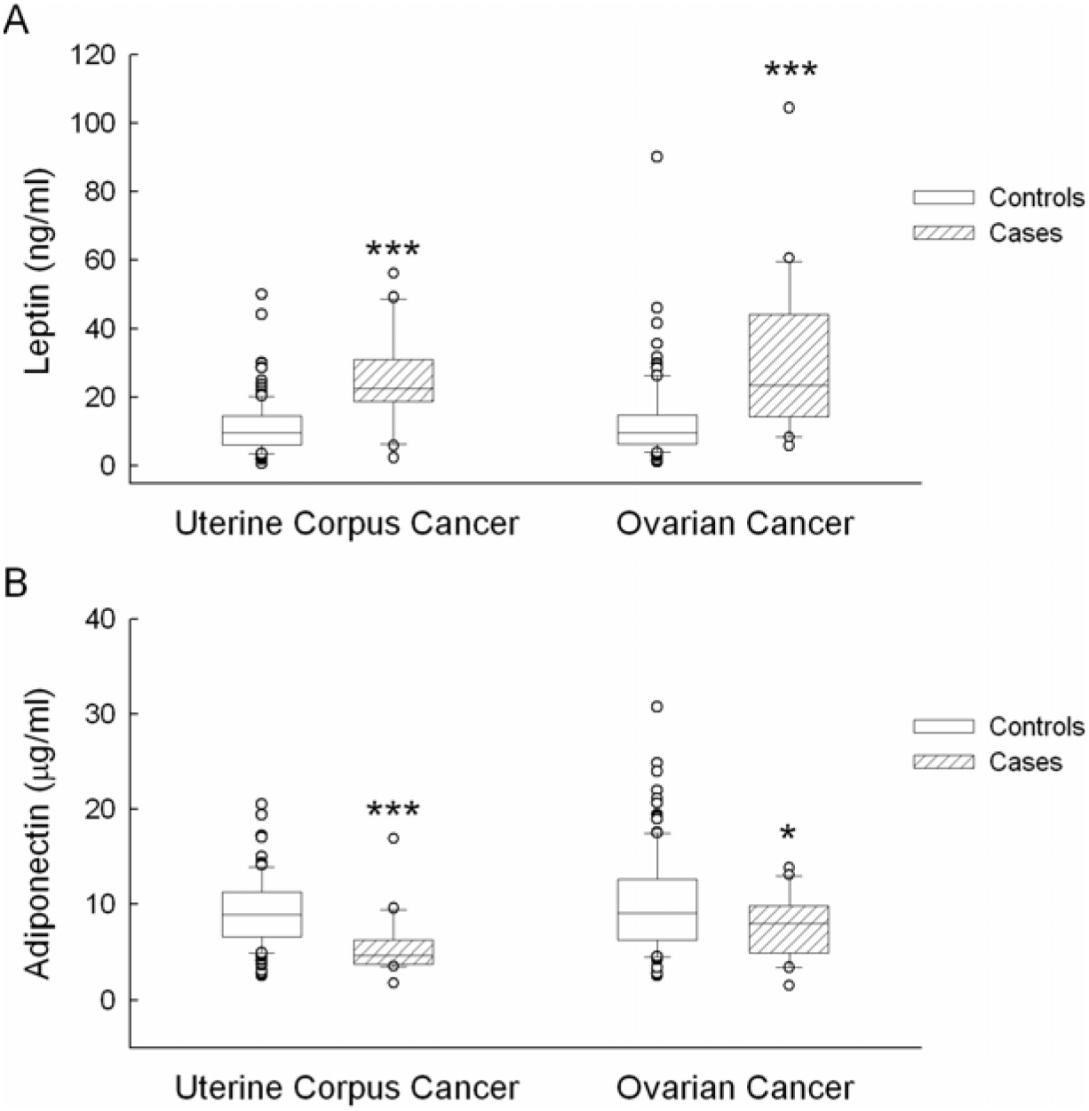
Box plot describing plasma adipokine levels in relation to gynecological cancers. **A.** Leptin levels in uterine corpus cancer cases (UCC, n = 20), ovarian cancer cases (OVC, n = 20) and their respective age-menopause–matched controls (n = 120 and 120, respectively). **B.** Adiponectin levels in uterine corpus cancer cases (n = 20), ovarian cancer cases (n = 20) and their respective age-menopause–matched controls (n = 120 and 120, respectively). *p<0.05 and ***p<0.001 vs. controls after adjusting for age, alcohol intake (for UCC model only), and years of endogenous estrogen exposure. Adipokine variables were log-transformed before the analysis for significance test.

In the nested case-control study, case subjects with incident gynecological cancer had a significantly higher level of leptin and a significantly lower level of adiponectin in plasma at enrollment as compared with control subjects (Figure 2). Leptin was median 22.53 ng/ml (interquartile range: 19.47–29.05 ng/ml) in UCC cases versus 9.81 (6.16–14.56) in the age- and menopause-matched controls, and 23.58 (14.92–42.61) in OVC cases versus 9.79 (6.50–14.74) in their matched controls. The corresponding adiponectin levels were 4.71 µg/ml (3.95–6.62 µg/ml) versus 8.92 (6.66–11.28) for UCC and 7.98 (5.21–9.60) versus 9.08 (6.37–12.61) for OVC. As shown in Table 5, after adjusting for age and risk covariates, subjects with leptin in the highest tertile 3 had an increased risk of incident gynecological cancer as compared with those in the lowest tertile 1 (OR = 10.68, 95% CI = 2.09–54.67, *p* = 0.005 and OR = 11.83, 95% CI = 1.40–100.11, *p* = 0.023 for UCC and OVC, respectively). On the other hand, subjects with baseline adiponectin in the tertile 3 had a decreased risk of subsequent gynecological cancer as compared with those in the tertile 1 (OR = 0.07, 95% CI = 0.01–0.62, *p* = 0.016 and OR = 0.31, 95% CI = 0.07–1.30, *p* = 0.108 for UCC and OVC, respectively). Worthy of note, the triglycerides effect on UCC risk turned out to be insignificant and was reduced by 72% when adiponectin was added into the regression model [(0.9932–0.2767)/0.9932, the β coefficient for triglyceride in the multivariate-adjusted model without adiponectin included and compared to the β coefficient for triglyceride in the model with adiponectin included]. Accordingly, the triglyceride effect on UCC risk was reduced by 22% after leptin was added into the model for adjustment [(0.9932–0.7729)/0.9932]. The BMI effect on OVC risk was reduced by 84% when leptin was further adjusted in the model [(0.8879–0.1387)/0.8879], and the BMI effect on OVC risk was reduced by 9% after adjusting for adiponectin [(0.8879–0.8082)/0.8879]. By contrast, with triglycerides added to the model, the leptin or adiponectin remained significantly associated with increasing UCC risk. By the same token, the association of leptin or adipoection with OVC risk was not substantially changed when BMI was entered into the regression model.

**Table 5:** Odds ratio (OR) with 95% confidence interval (CI) of risk factors and plasma adipokine levels in the prediction of developing gynecological cancer.

	Uterine corpus cancer (UTC)		Ovarian cancer (OVC)	
Characteristics	OR	(95% CI)	OR	(95% CI)
*Age-adjusted model* a				
BMI	0.78	(0.24–2.55)	2.41	(0.89–6.49)
Triglyceride	2.73	(1.00–7.49)	0.88	(0.31–2.52)
Endogenous estrogenexposure	1.76	(0.80–3.87)	1.31	(0.70–2.43)
Leptin				
Tertile 2^b^	0.53	(0.05–6.07)	2.69	(0.27–27.15)
Tertile 3	8.76	(1.86–41.17)**	12.95	(1.64–102.52)*
Adiponectin				
Tertile 2	0.17	(0.05–0.62)**	0.92	(0.32–2.66)
Tertile 3	0.07	(0.01–0.52)**	0.29	(0.07–1.15)
*Multivariate-adjusted model for leptin study*				
BMI	0.30	(0.08–1.09)	1.15	(0.38–3.47)
Triglyceride	2.17	(0.69–6.85)	0.80	(0.26–2.45)
Endogenous estrogenexposure	1.68	(0.73–3.89)	1.10	(0.57–2.14)
Leptin				
Tertile 2	0.46	(0.04–5.50)	2.80	(0.27–28.65)
Tertile 3	10.68	(2.09–54.67)**	11.83	(1.40–100.11)*
*Multivariate-adjusted model for adiponectin study*				
BMI	0.59	(0.17–2.09)	2.24	(0.82–6.13)
Triglyceride	1.32	(0.42–4.18)	0.79	(0.26–2.40)
Endogenous estrogenexposure	1.76	(0.74–4.20)	1.10	(0.57–2.11)
Adiponectin				
Tertile 2	0.15	(0.04–0.61)**	0.83	(0.27–2.53)
Tertile 3	0.07	(0.01–0.62)*	0.31	(0.07–1.30)

a Age effect was assessed as a continuous variable by year, and the risk associated with years of endogenous estrogen exposure was estimated by 5-year increment. The other variables were categorized as BMI (body mass index) ≥27 versus <27 kg/m^2^ (reference), and triglycerides ≥150 versus <150 mg/dL (reference).

b Tertile 1 as reference with OR = 1.00 for leptin and adiponectin variables.

*p<0.05; **p<0.01.

## Discussion

Results from this prospective study confirm that Taiwan is a low-incidence area for non-viral gynecologic cancers, namely UCC and OVC. The incidences are similar to recent reports for Japanese and Chinese women populations [29], [30], but are a half lower risk than those for British women reported in a recent cohort study, indicating 45.0 cases of UCC and 38.9 cases of OVC per 100,000 person-years [10]. In association analysis of our study, results showed that alcohol intake, serum triglycerides, and years of endogenous estrogen exposure are significant risk factors for developing UCC among Taiwanese women. By contrast, elevated BMI level was a significant predictor for the development of OVC in the women cohort. Analysis results further demonstrated a significant association of high plasma leptin levels and low plasma adiponectin levels with an increased risk of developing UCC, as well as high plasma leptin levels with an enhanced risk for becoming OVC in this cohort. The circulating adipokine levels may contribute to, at least in part, the link between adiposity factors and the development of gynecological cancer in this low-incidence population.

### Risk Factors for UCC Incidence

Alcohol intake was the strongest predictor of UCC risk in our data. Comparatively, few studies have assessed the association between alcohol intake and the development of UCC; the results of existing studies offered little support for an association. A recent large prospective cohort study reported a significantly increased risk of UCC with increasing alcohol consumption (≥2 drinks/day) [8], whereas a later cohort study found no clear association between alcohol intake and UCC risk [10]. Daily alcohol use has been related to elevated levels of circulating estrogens in postmenopausal women [31], indicating a plausible mechanism for alcohol intake in UCC development. Although our analysis results were consistent with some published reports and supported a positive association, additional studies of a large sample of Asian women are needed to confirm our findings.

Among the risk factors for becoming UCC in Taiwanese women, serum triglyceride level was the most important risk factor for preventing the cancer risk in terms of PAF if the triglyceride level was lowered to normal range. Our finding of a positive association with triglycerides is consistent with the results from two recent epidemiologic studies, one involving Nordic women in prospective design and the other Chinese women in case-control setting [9], [32]. Notably, some researchers argued that the effect of serum triglycerides may be explained by BMI levels [9], [11]. In our data, the correlation of triglyceride levels with BMI was 0.35 (Spearman correlation, *p*<0.001). Even though BMI level along with other risk factors were adjusted for simultaneously in our regression analysis, triglycerides was still positively associated with UCC risk at a significant level (*p* = 0.008, Table 4). Alternatively, the association of triglycerides with UCC risk may be explained by the years between menopause and cancer diagnosis. We therefore performed a sensitivity analysis that were limited to the women who had no menopause at the time of baseline survey, and obtained a similar effect of triglycerides on UCC risk (HR = 2.47, 95% CI = 1.11–5.51, *p* = 0.027). We speculated that some mechanisms specifically related to triglycerides may influence the UCC carcinogenesis among Taiwanese women, at least in premenopausal women. In mouse model, adiponectin has been shown to involve in the transport and combustion of free fatty acid (FFA), thereby reduces serum FFA and triglycerides level [33]. Decreased triglycerides may in turn contribute to the improved insulin signal transduction and reverse insulin resistance of obese mice [33]. Although we do not have evidence supporting a role of insulin in UCC development in the present study, we propose that the effect of triglycerides may be attributed to the low adiponectin levels of the UCC cases. This proposition is supported by the data shown in Table 5 that the triglycerides effect on UCC risk became less significant in multivariate models after adjustment of adiponectin levels.

Our initial results found no significant association between obesity (BMI ≥27 kg/m^2^) and the risk of UCC in the study cohort (Tables 3 and 4). One possible reason is that the number of incident UCC cases was too small and therefore the analysis of BMI effect was lacking an adequate statistical power. Alternatively, BMI might play a less important role in contrast to triglycerides on the development of UCC in this women population. Notwithstanding, by examining the lifetime cumulative incidence on attained age during follow-up, we still observed that the UCC cases were more likely to occur in the group of women with an elevated BMI (≥27 kg/m^2^) than in the group of women with normal BMI (<27 kg/m^2^) at all ages throughout an individual’s lifetime (Fig. 1.c).

### Risk Factors for OVC Incidence

In Western countries, white women with obesity showed only weak or moderate risk for OVC as compared with those of normal weight [34]. Olsen et al conducted a meta-analysis of 28 population-based studies and found the pooled RR of OVC for obesity was 1.30 (95% = 1.12–1.50); the pooled risk was even weaker among cohort studies (RR = 1.12) than case-control studies (OR = 1.49) (Olsen CM 2007 Eur J Can). A pooled analysis of 12 cohort studies reported an overall RR of 1.03 (95% = 0.86–1.22), with a stronger effect among premenopausal women (RR = 1.72, 95% = 1.02–2.89), but a weaker effect among postmenopausal women (RR = 1.07, 95% = 0.87–1.33) [35]. In our data, the magnitude of association for mixed pre-and post-menopausal women was higher (HR = 2.90, 95% = 1.30–6.46) than those previously reported for study subjects of North America and Europe in the literature. Comparatively, Asian women are less frequent users of exogenous hormones than Western women, which might mask a stronger positive relationship between BMI and OVC risk than achieved for the Western women. Our data further showed that the impact of obesity on OVC risk amounting to a 2 to 3-fold high risk remained similar across all ages throughout the lifetime of Taiwanese women. In other words, no apparent differential effect of BMI was observed between pre- and post-menopausal ages. To explore a biological mediator for this association, we analyzed data and found that the strong association of obesity with OVC risk was attenuated (OR from 2.41 to 1.15) after adjustment of leptin levels, suggesting that the BMI effect may be explained by the high leptin levels in the OVC cases.

Results of the present study did not support a sex hormonal etiology of OVC risk for Taiwanese women. Endogenous hormones have been proposed to play a role in OVC carcinogenesis. However, a clear association with reproductive factors and identification of their underlying hormonal components are still lacking [12], [13]. In recent literature reviews, hereditary components have been also suggested to play an important role in increasing OVC risk [12], [13]. In our data, the prevalence of a family history of OVC was low, suggesting that the observed null association of endogenous hormone exposure with OVC risk in this cohort was less likely explained by the genetic predisposition. The results of our study on an Asian population did not support a role of hormonal etiology in the development of OVC.

### Adipokines and Gynecological Cancer Risk

Leptin and adiponectin are the two most well studied adipokines produced by adipose tissue in relation to obesity-related cancers, including breast, colon, hepatic, and endometrial cancer [18]. Clinical studies have described the association of a high leptin and a low adiponectin level in patients with endometrial cancer, and predicted a poor prognosis of these patients [36], [37]. In case-control studies, endometrial cancer has been associated with high levels of leptin and low levels of adiponectin in a Greek and Japanese population [19], [20]. To date, two of four prospective studies support the results from the case-control studies, yet predominantly included women of European descent [21], [22]. Our data on Asian women, derived from a nested case-control study with supporting in cause-effect relationship, are consistent with these study results. Our results also support the findings in various cancer cell lines [38], [39] indicating that leptin and adiponectin exert opposing effect in UCC carcinogenesis. Furthermore, we demonstrated, for the first time, that higher leptin levels assayed years long before cancer diagnosis were significantly associated with an increased risk of OVC. This finding adds to the previous studies noted in patients of obesity-related cancers and suggests that hyperleptinemia may as well predict the development of OVC on a period of follow-up in apparently normal subjects.

### Strengths and Limitations of the Study

A major strength of this study is the prospective design, thus an inverse causation bias resulting from pre-existing conditions was unlikely to occur. Furthermore, our study subjects were enrolled from seven townships representative of Taiwan inhabitants, allowing us to provide risk estimates and prevention strategies in gynecologic cancers for the general population in Taiwan. The quality of the nation-wide cancer registry guaranteed accuracy in our results and completeness in identification of incident cases of cancer [2].

Small number of sample cases is the first limitation of the current study. Consequently, the observed significant findings of the study may be due to chance. Although incident cases were few, the number of the individuals in the reference groups was large, allowing adequate precision in estimates. Secondly, our findings were based on a single baseline measure of adiposity factors and other risk factors. Weight gains or lifestyle changes during the follow-up period can not be evaluated. Thirdly, for the calculation of total years of endogenous estrogen exposure, we did not take the duration of breastfeeding (estrogen interruption) into account. Given the close negative association of breastfeeding with UCC risk, the risk estimates for the association of total years of endogenous estrogen exposure in our study may likely have been underestimated. Finally, we included the cases of study cancers as a whole, rather than as histological subtypes in analysis, because of the small number of incident cases. The possibility of disease heterogeneity related to risk factors cannot be examined in this study. However, the association between obesity and OVC subtypes has not been consistently reported in the literature. Some reported that obesity was a significant risk factor only for endometrioid type of ovarian cancer [40], while others reported an increased risk of all invasive cancers except for high grade serous type [41], [42]. In our study, histological subtypes of OVC included serous (n = 14), mucinous (n = 4), clear cell (n = 2), endometrioid (n = 1), and other specified carcinoma (n = 9). Regarding UCC risk, Lindemann et al reported that obesity increases the cancer risk as a whole entity with a stronger effect for endometrioid adenocarcinoma [9]. In our study, patients with endometrial cancers comprised 84% of total UCC cases, including 29 endometrioid adenocarcinoma and 3 adenosquamous carcinoma. The small number of cases in the study limits our ability to evaluate the relationships between study cancers and risk factors by histological subtypes.

### Conclusions

Our findings provided evidence that, in addition to estrogen exposure, other risk factors such as alcohol intake and serum triglycerides may also be involved in UCC carcinogenesis. By contrast, obesity is the dominant predictor for OVC risk among the study cohort. Our data also suggest that circulating leptin and adiponectin may mediate the link of triglycerides and obesity, respectively, to UCC and OVC risk. Since triglyceride levels and obesity are modifiable in health management, our study results support recommending specific lifestyle modifications in individuals to reduce gynecological cancer risk.

## Supporting Information

Figure S1 **Relationship between plasma adipokine levels and BMI values in a subcohort (n = 546) out of the original CBCSP-HPV cohort.** **p<0.01 and ***p<0.001 for comparisons between the higher tertile 2 or 3 vs. the lowest tertile 1.
(TIFF)
