# Supplementary figures and images for: A Prospective Study of Gynecological Cancer Risk in Relation to Adiposity Factors: Cumulative Incidence and Association with Plasma Adipokine Levels
Source: PLoS One. 2014 Aug 12;9(8):e104630. doi: 10.1371/journal.pone.0104630 (PMC4130554; doi:10.1371/journal.pone.0104630)

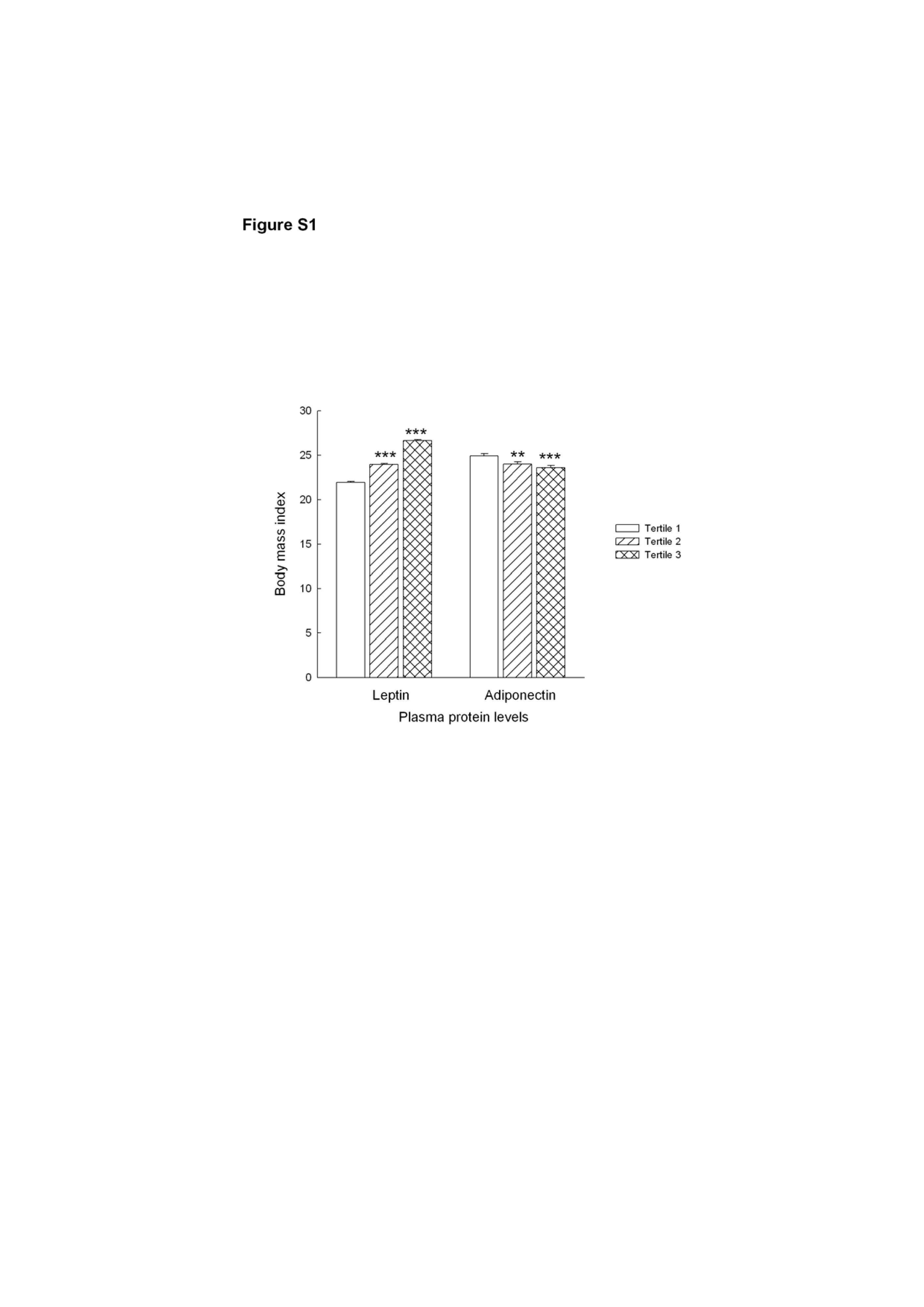

Supplement: Figure S1 — Relationship between plasma adipokine levels and BMI values in a subcohort (n = 546) out of the original CBCSP-HPV cohort. **p<0.01 and ***p<0.001 for comparisons between the higher tertile 2 or 3 vs. the lowest tertile 1. (TIFF) [file pone.0104630.s001.tiff]
